# WormPaths: *Caenorhabditis elegans* metabolic pathway annotation and visualization

**DOI:** 10.1093/genetics/iyab089

**Published:** 2021-06-12

**Authors:** Melissa D Walker, Gabrielle E Giese, Amy D Holdorf, Sushila Bhattacharya, Cédric Diot, Aurian P García-González, Brent B Horowitz, Yong-Uk Lee, Thomas Leland, Xuhang Li, Zeynep Mirza, Huimin Na, Shivani Nanda, Olga Ponomarova, Hefei Zhang, Jingyan Zhang, L Safak Yilmaz, Albertha J M Walhout

**Affiliations:** Program in Systems Biology and Program in Molecular Medicine, University of Massachusetts Medical School, Worcester, MA 01609, USA

**Keywords:** *C. elegans*, metabolism, metabolic pathways, pathway visualization, pathway enrichment analysis

## Abstract

In our group, we aim to understand metabolism in the nematode *Caenorhabditis elegans* and its relationships with gene expression, physiology, and the response to therapeutic drugs. Visualization of the metabolic pathways that comprise the metabolic network is extremely useful for interpreting a wide variety of experiments. Detailed annotated metabolic pathway maps for *C. elegans* are mostly limited to pan-organismal maps, many with incomplete or inaccurate pathway and enzyme annotations. Here, we present WormPaths, which is composed of two parts: (1) the careful manual annotation of metabolic genes into pathways, categories, and levels, and (2) 62 pathway maps that include metabolites, metabolite structures, genes, reactions, and pathway connections between maps. These maps are available on the WormFlux website. We show that WormPaths provides easy-to-navigate maps and that the different levels in WormPaths can be used for metabolic pathway enrichment analysis of transcriptomic data. In the future, we envision further developing these maps to be more interactive, analogous to road maps that are available on mobile devices.

## Introduction

Metabolism can be broadly defined as the total complement of reactions that degrade and synthesize biomolecules to produce the biomass and generate the energy organisms need to grow, function, and reproduce. Metabolic reactions function in metabolic pathways that are interconnected to form the metabolic network. In metabolic networks, the nodes are metabolites and the edges are conversion and transport reactions carried out by metabolic enzymes and transporters. 

Genome-scale metabolic network models provide mathematical tools that are invaluable for the systems-level analysis of metabolism. Such models have been constructed for numerous organisms, including bacteria, yeast, the nematode *Caenorhabditis elegans*, and humans (Yilmaz and Walhout 2017). Metabolic network models are extremely useful because they can be used with flux balance analysis to derive specific insights and hypotheses. For example, gene expression profiling data can be used to gain insight into metabolic network activity at pathway, reaction, and metabolite levels under different conditions or in particular tissues (Machado and Herrgard 2014; Yilmaz and Walhout 2016; Opdam *et al.* 2017; Yilmaz *et al.* 2020).

Visualizing the metabolic pathways that together comprise the metabolic network of an organism is extremely useful to aid in the interpretation of results from different types of large-scale, systems-level studies such as gene expression profiling by RNA-seq, phenotypic screens by RNAi or CRISPR/Cas9,or genetic interaction mapping. Several resources are available online for the visualization and navigation of metabolic pathways. Probably the most widely used is the Kyoto Encyclopedia of Genes and Genomes (KEGG), a platform that provides pan-organism annotations and metabolic pathway maps (Kanehisa *et al.* 2015). Other online resources include MetaCyc (Caspi *et al.* 2014), BRENDA (Chang *et al.* 2015), and REACTOME (Joshi-Tope *et al.* 2005). While all of these platforms are extremely useful resources for metabolic pathway mapping, enzyme classification, and pathway visualization, they can have incomplete or incorrect pathway and enzyme information due to a lack of extensive manual curations for specific organisms. As a result, map navigation can be rather non-intuitive.

Over the last five decades or so, the free-living nematode *C. elegans* has proven to be an excellent genetic model to gain insights into a variety of biological processes, including development, reproduction, neurobiology/behavior, and aging (Corsi *et al.* 2015; Lemieux and Ashrafi 2016; Nigon and Felix 2017). More recently, *C. elegans* has emerged as a powerful model to understand basic metabolic processes (Watson and Walhout 2014; Rashid *et al.* 2020). *C. elegans* is a bacterivore that can be fed different bacterial species and strains in the lab (MacNeil and Walhout 2013; Yilmaz and Walhout 2014). Numerous studies have begun to shed light on the metabolic mechanisms by which different bacterial diets can affect the animal’s metabolism (Larsen and Clarke 2002; Coolon *et al.* 2009; Gusarov *et al.* 2013; MacNeil *et al.* 2013; Watson *et al.* 2013; Virk *et al.* 2016; Zhang *et al.* 2019). For instance, we have discovered that when fed a diet low in vitamin B12, *C. elegans* adjusts the two metabolic pathways that rely on this cofactor. Specifically, it rewires propionate degradation by transcriptionally activating a propionate shunt and upregulates Methionine/S-adenosylmethionine cycle genes to adjust cycle activity (Watson *et al.* 2014; 2016; Bulcha *et al.* 2019; Giese *et al.* 2020). To enable more global analyses of *C. elegans* metabolism, we previously reconstructed its first genome-scale metabolic network model (Yilmaz and Walhout 2016). The recently updated version of this model includes 1314 genes, 907 metabolites, and 2230 reactions and is referred to as iCEL1314 (Yilmaz *et al.* 2020). Information about this network and all the components involved is publicly available on our WormFlux website (http://wormflux.umassmed.edu).

Over time, we found that we were missing metabolic pathway maps that are easy to navigate and that can be used to help interpret results from phenotypic screens and gene expression profiling experiments. We used KEGG pathways, which provide generic, non-organism-specific visualizations, as a starting point to redraw maps of *C. elegans* metabolism on paper to help us interpret our data. In KEGG, enzymes are indicated by Enzyme Commission numbers and maps are colored with those enzymes predicted to be present in an organism of interest; however, organism-specific pathways cannot be extracted. Furthermore, many of these maps contain incorrect or partially correct reactions for *C. elegans*. We found that redrawing pathway maps that contain information about metabolites, genes encoding the proteins that catalyze metabolic reactions or transport metabolites between cells or cellular compartments, molecular structures, and cofactors was very helpful to our studies (Watson *et al.* 2016; Bulcha *et al.* 2019; Giese *et al.* 2020).

Here, we present WormPaths, a web-based collection of standardized metabolic pathway maps for *C. elegans*. In total, WormPaths contains 62 maps covering major metabolic pathways (glycolysis/gluconeogenesis, TCA cycle, *etc*.), amino acid metabolism, and pathways fundamental to *C. elegans* physiology (collagen biosynthesis, ascaroside biosynthesis, propionate degradation, *etc*.). Each map connects to other pathways, thereby covering the entire iCEL1314 network. Importantly, the network was expanded by adding reactions and genes found in the literature that were heretofore missed. Maps were carefully curated, hand-drawn, and then visualized in a standardized Scalable Vector Graphics (SVG) format, which allows interactive usage in web applications. WormPaths annotations and maps are publicly available on the WormFlux website (http://wormflux.umassmed.edu). Our careful gene-to-pathway annotations at different levels (see Results) enable statistical enrichment analyses. Finally, our maps may provide a useful format for the drawing of metabolic pathway maps in other organisms. In the future, we envision further refining the maps through detailed literature reviews and experiments.

## Materials and methods

### Design of pathway maps

The design of pathway maps aimed at capturing and visualizing metabolic functions in such a way that would be broadly useful for both statistical analyses and navigation purposes. The starting point for pathway definitions was the pathway annotations of reactions and genes of iCEL1314 in WormFlux and in KEGG. Existing pathways were then split and/or modified such that the functional resolution of pathways was increased without disrupting the coherence of reactions, while the number of overlapping reactions was minimized. For example, the valine, leucine, and isoleucine degradation pathway (one map in KEGG) was first divided into three maps to increase pathway resolution: valine degradation, leucine degradation, and isoleucine degradation. Then, a reaction that existed in the original pathway that converts propionyl-CoA to methylmalonyl-CoA (*i.e*., RM01859 in iCEL1314 and R01859 in KEGG) was removed from valine degradation and isoleucine degradation maps to avoid a redundant overlap with propionate degradation, where this reaction defines a critical step. In KEGG, R01859 is associated with glyoxylate and dicarboxylate metabolism in addition to valine, leucine, and isoleucine degradation and propionate metabolism, thus appearing in three places. However, propionyl-CoA to methlymalonyl-CoA conversion is clearly the first step of canonical propionate degradation.

Typically, pathways were designed to start or end with three types of metabolites: (i) the main substrate or product by definition (*e.g.*, histidine is the starting point in histidine degradation, and collagen is the endpoint in collagen biosynthesis), (ii) a connection to other pathways (*e.g.*, valine degradation ends with propionyl-CoA through which it is connected to propionate metabolism), and (iii) an endpoint that can be transported to or from extracellular space (*e.g.*, histamine is produced in histidine degradation pathway and exported). The connections of terminal metabolites to other pathways are indicated in maps by clickable pathway boxes as in KEGG, unless the metabolite is associated with more than two other pathways. When a terminal metabolite is not associated with any other pathway, a proper transport that explains the source or fate of the metabolite is included. If a transport is not available either, then it follows that the metabolite is associated with reactions not included in WormPaths maps yet, which is indicated by a box labeled “other”. In any case, the number of pathways and the types of transports (cytosol-extracellular space or mitochondria-cytosol) a metabolite is associated with are indicated by colored squares and circles, respectively, as shown by a legend appended to every map. Furthermore, clicking a metabolite brings the page of that metabolite in WormFlux, which shows all pathways and reactions it is associated with. Thus, information about the pathway associations and transportability of, not just terminals, but every metabolite in a pathway, is reachable from the pathway map.

### Illustration of pathway maps

Draft maps were drawn as SVG files in the open-source vector graphics editor Inkscape (http://inkscape.org) following a template (Supplementary Figures S1 and S2). Genes from each map were extracted from the SVG files and cross-referenced to the master levels spreadsheet (Supplementary Table S1). After correction of errors, the final SVG maps were wrapped with HTML format and uploaded to the WormFlux website (http://wormflux.umassmed.edu, last accessed June 18, 2021). Maps were blended with WormFlux pages and made interactive using PHP language for server-side processes (*e.g.*, search) and JavaScripting language for the client-side actions (*e.g.*, metabolite image display).

### Pathway enrichment analysis

To facilitate pathway enrichment analysis (PEA), we developed an interactive webtool in the WormFlux website (http://wormflux.umassmed.edu/WormPaths/pea.php, last accessed June 18, 2021). This tool takes a list of genes from the user as the input. First, the overlap between the input and the background (universal) set, *i.e.*, the entire gene list of iCEL1314, is determined. This overlap defines the metabolic hits. For each gene category at each level, the strength and significance of the intersection between category genes and metabolic hits are evaluated. The strength is the enrichment score calculated as *k*/*n*, where *k* is the number of genes in the intersection set, and *n* stands for the total number of genes in that category. The significance was based on the hypergeometric test performed using the **hypergeom** function in **scipy** package of Python 2.7. Briefly, a hypergeometric distribution is derived for the size of the intersection set between the category genes and the metabolic hits using *hypergeom*(*M*, *N*, *n*), where *M* is the size of the universal set, which is 1314, and *N* is the number of metabolic hits. Then, the probability mass function of this distribution is used to calculate the enrichment *p*-value as the sum of probabilities for *k* and greater integers, which corresponds to the total probability of having *k* or more genes in the intersection. Similarly, the depletion *p*-value is calculated as the total probability of having *k* or less genes in the intersection. The PEA tool provides an interactive color-coded table that illustrates enrichment scores and enrichment or depletion *p*-values for every category at every level. The results are also provided in greater detail as a downloadable tab-separated text file. Importantly, a multiple hypothesis testing correction is not done by PEA to avoid the underestimation of enrichment strength. The users are encouraged to apply Bonferroni correction when searching enrichments or depletions at a significance level of *α*, such that *p *< *α/n_test* must be satisfied, where *n_test* is the number of tests (*e.g.*, the number of categories at a level of interest). A workflow is shown in Supplementary Figure S3.

### Metabolite structures

Out of the 907 metabolites in iCEL1314, 777 are represented in WormPaths maps by abbreviations that are linked to images with metabolite name, formula, and structure. The image of a metabolite is displayed when its abbreviation is hovered over. Names and formulas follow from iCEL1314 (Yilmaz *et al.* 2020). Structures were based on mol file representations (Dalby *et al.* 1992) or hand drawings. Mol files were readily obtained from KEGG (Kanehisa *et al.* 2015) for 559 metabolites, and from other public resources including Virtual Metabolic Human Database (Noronha *et al.* 2019), PubChem, and ChEBI for 43 more. All mol files were converted to PNG format using Open Babel (O'Boyle *et al.* 2011). The structures of 160 metabolites were created based on mol files and shapes of similar molecules using a commercial vector-based graphics software when necessary. These drawings were also saved in PNG format. No definitive structures were found for the remaining 15 metabolites (mostly proteins) for which a “Structure not available” sign was used instead of a molecular structure. Finally, each structure image was stacked with the corresponding metabolite name and formula using Inkscape to obtain the images of metabolites displayed in WormPaths.

### Data availability

All pathway annotations from this study are available in the Supplementary Tables S1, S3–S5 and are downloadable from the WormFlux website (http://wormflux.umassmed.edu/download.php, last accessed June 18, 2021). All pathway maps are available in WormFlux (http://wormflux.umassmed.edu/WormPaths/wormpaths.php) and can be used interactively to visualize information related to metabolites, genes, reactions, and pathways in this website. Each pathway map can be downloaded in PNG, PDF, and SVG formats from the corresponding pathway page.

Supplementary material is available at *GENETICS* online.

## Results

### Assigning *C. elegans* metabolic genes to pathways at different levels

**Figure 1:**
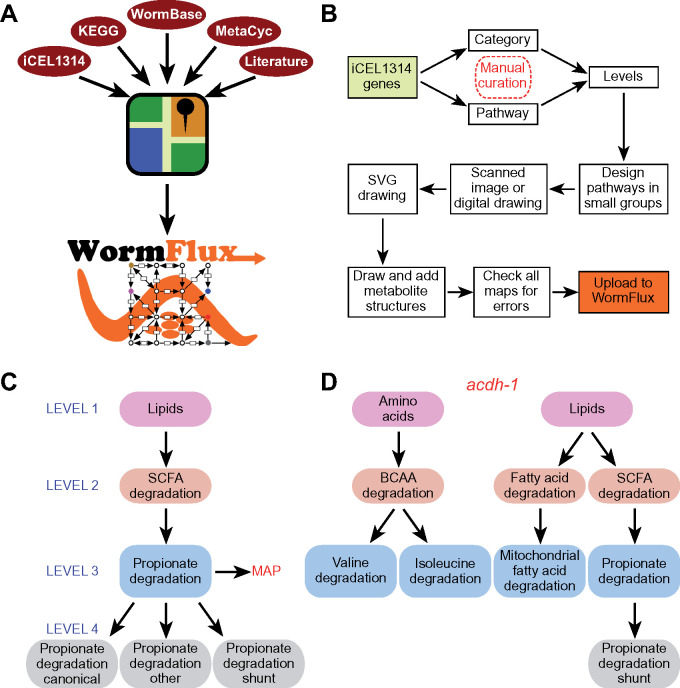
WormPaths annotation of *C. elegans* metabolic genes. (A) Cartoon outlining resources used to generate WormPaths. (B) Pipeline of gene to pathway/category annotations and map construction. (C) Example of pathway-centered WormPaths annotations. (D) Example of gene-centered WormPaths annotations. BCAA, branched-chain amino acids; SCFA, short chain fatty acids; SVG, scalable vector graphics.

To generate WormPaths, we built on available resources, most notably the iCEL1314 metabolic network model (Yilmaz *et al.* 2020), KEGG (Kanehisa *et al.* 2015), MetaCyc (Caspi *et al.* 2014), WormBase (Harris *et al.* 2020), and literature searches (Figure 1A). Briefly, we manually curated each of the 1314 genes present in the iCEL1314 model and assigned them to one or more biochemical pathway (see Materials and methods). In addition, we used a “category” annotation for metabolic genes that best fit in groups or enzyme categories rather than specific biochemical pathways (Figure 1B). Examples of categories include the 40 iCEL1314 genes in complex I of the electron transport chain, guanylate cyclases that convert guanosine triphosphate to cyclic guanosine monophosphate, and vacuolar ATPases that maintain proton gradients across organellar plasma membranes. Because all metabolic pathways are connected into a metabolic network and some pathways are embedded, or nested, into larger pathways, we decided to annotate *C. elegans* metabolic pathways at different levels. Categorizing genes into pathways at different levels enables enrichment analyses at different levels of resolution (see below). Level 1 includes the broadest assignment to ten annotations: amino acids, carbohydrates, cofactors and vitamins, energy, lipids, nucleotides, one-carbon cycle, reactive oxygen species, other amino acids, and other (Supplementary Table S1). Levels 2, 3, and 4 further refine pathways within Level 1 annotations. For instance, the propionate shunt (Watson *et al.* 2016) (Level 4) is part of propionate degradation (Level 3), which is part of short-chain fatty acid degradation (Level 2), which is part of lipids (Level 1) (Figure 1C, Supplementary Table S1). Altogether, there are 10 groups of pathways or categories at Level 1, 61 groups at Level 2, 79 groups at Level 3, and 85 groups at Level 4. Not all Levels 2 or 3 can be further subdivided, and therefore there is redundancy at the higher levels (3 and 4) (Supplementary Table S1). For each pathway, we decided as a group which level would be most useful for visualization as a map, and a team of two lab members worked together to design and draw a draft map (Supplementary Table S2). For example, Level 2 branched-chain amino acid degradation can be subdivided into three maps at Level 3: isoleucine, leucine, and valine degradation, each of which is visualized separately. Another example is methionine metabolism (Level 3), which can be further refined to methionine salvage and the methionine/S-adenosylmethionine cycle (Level 4). Other amino acids need no further categorization and maps are drawn at Level 2, such as histidine and lysine degradation.

In WormPaths, 32% of genes are annotated to multiple pathways. While many genes do in fact act in multiple pathways, others may be annotated to multiple pathways because gene–protein-reaction annotations are based on homologies with known enzymes, and the exact participation of each gene in different pathways cannot be resolved without experimentation. For instance, the acyl-CoA dehydrogenase-encoding gene *acdh-1* is annotated to different degradation reactions in amino acid and lipid metabolism (Figure 1D, Supplementary Tables S3–S5). However, only its role in the propionate shunt has been experimentally characterized (Watson *et al.* 2016). Importantly, its close paralog *acdh-2* is annotated to the same pathways but was experimentally shown *not* to be involved in the propionate shunt (Watson *et al.* 2016). Future biochemical and genetic studies are needed to disentangle which enzymes can catalyze multiple reactions, and which are specific to individual reactions.

### WormPaths maps—visualization and navigation

After map level assignments and pathway design, maps were sketched digitally or by hand and electronically uploaded to Google Docs for sharing followed by manual conversion to SVG format, an Extensible Markup Language (XML)-based vector image format for general useability on the Internet by both individual users and computer programs. Metabolite structure images for all products and reactants were downloaded from KEGG and other resources (see Materials and methods) and some that were not available were hand drawn. All reactions on the SVG maps were manually verified and checked for errors. Maps were then uploaded to the WormFlux webpage, where they are available in a drop-down list. All maps are searchable and clickable. For example, a search for the gene *metr-1* will result in the WormFlux gene page for *metr-1*, which has links to the methionine/S-adenosylmethionine cycle and folate cycle pathways, each of which brings the corresponding map with the *metr-1* gene highlighted (Supplementary Figure S4). In reverse, clicking on a gene in any map leads to the associated WormFlux page, where key identifiers and reactions in which the gene is involved are listed. The same is true when searching and clicking metabolites.

**Figure 2:**
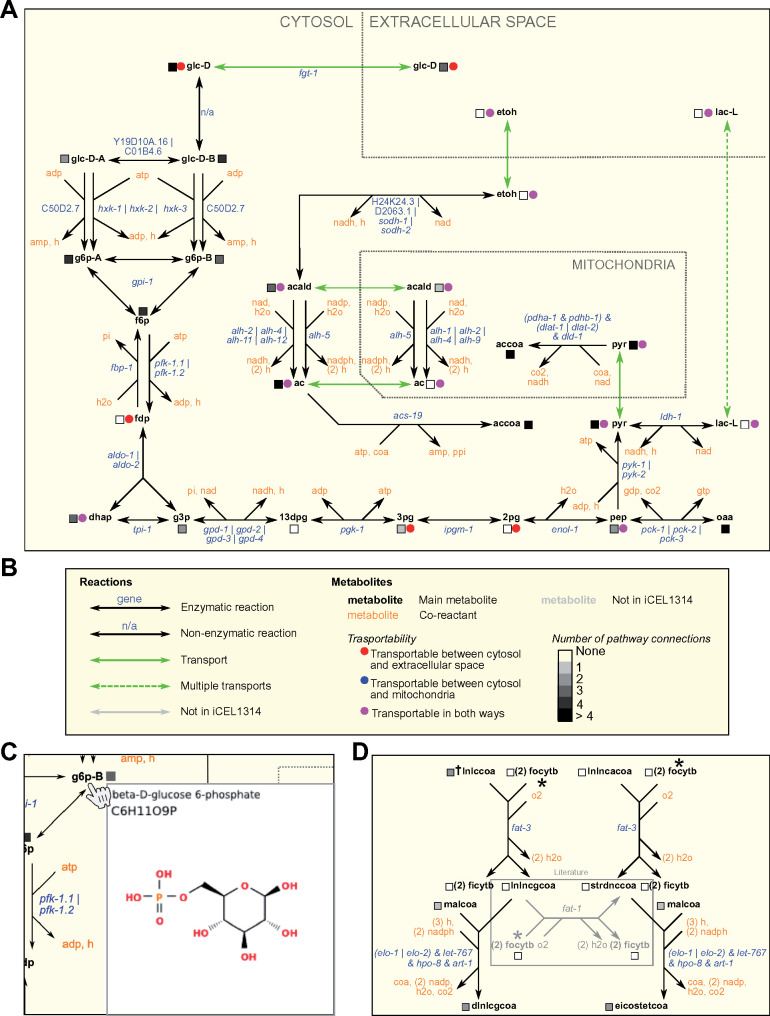
WormPaths examples. (A) WormPaths map of glycolysis/gluconeogenesis. (B) The key to the reactions, metabolite transportability, and number of pathway connections that appears on the WormPaths website. (C) An example of a web pop-up window from glycolysis/gluconeogenesis that shows the metabolite structure of beta-D-glucose 6-phosphate upon hovering the cursor over g6p-B. (D) Example of a literature-curated reaction highlighted in the gray box.

In total, WormPaths provides 62 maps of *C. elegans* metabolic pathways that connect into the larger iCEL1314 network. Figure 2A shows an example of the WormPaths map for glycolysis/gluconeogenesis. This is a Level 2 map that is part of carbohydrates (Level 1). The keys for different types of reactions are provided in Supplementary Figures S1 and S2. In metabolic networks, nodes are metabolites and edges are the reactions in which these metabolites are converted into one another, or transported between cellular compartments, or between the cell and the extracellular environment. The edges in these maps are black for enzymatic reactions and green for transport reactions (Figure 2B). The genes encoding the enzymes predicted to catalyze the reactions are indicated in blue, and co-reactants are indicated in orange (Figure 2A). Some reactions have multiple alternative genes associated with them. These “OR” genes are separated by a vertical bar (|). For example, in glycolysis/gluconeogenesis the interconversion between phosphoenolpyruvate (pep) and oxaloacetate (oaa) is associated with *pck-1*, *pck-2*, or *pck-3* (Figure 2A). None of these genes is associated with any other reaction and therefore they may function in different conditions or in different tissues (Yilmaz *et al.* 2020). Indeed, at the second larval (L2) stage, two of these three genes show very distinct tissue expression patterns, while the mRNA for the third gene (*pck-3*) was undetectable (Supplementary Figure S5) (Cao *et al.* 2017). For edges where multiple enzymes together catalyze a reaction, an ampersand (&) is used to indicate “AND” genes. For example, *pdha-1* and *pdhb-1* are both required in the pyruvate dehydrogenase complex that catalyzes the conversion of pyruvate (pyr) into acetyl-CoA (accoa) (Figure 2A).

For metabolite names both in WormPaths (Figure 2A) and in WormFlux (Yilmaz and Walhout 2016), we used Biochemical Genetic and Genomic (BiGG) database abbreviations where available (Schellenberger *et al.* 2010). The transportability of metabolites between subcellular compartments is indicated by a colored circle (Figure 2B), and the number of pathways connected between each metabolite is indicated by a grayscale square. When metabolites are hovered over by the cursor, the full name, formula, and chemical structure of the metabolite are displayed in an image that pops up (Figure 2C). For many transport reactions, the transporter is not yet known and only few have associated genes, or the transport gene is not part of the iCEL1314 metabolic model. We found that, by having multiple people manually evaluate different metabolic genes and pathways, the iCEL1314 metabolic model can be further improved. For example, we found that the conversion of γ-linolenoyl-CoA (lnlncgcoa) to stearidonyl-CoA (strdnccoa) by *fat-1* was missing from the model even though this reaction is described in the literature (Figure 2D) (Watts 2016). Using a combination of KEGG, WormBase, and literature searches, we added six genes to the maps that are not in the iCEL1314 model: two to new reactions and four to existing reactions. We also added 25 genes from the iCEL1314 model to 16 reactions: 5 existing and 11 new. Finally, we found three pathway connections not identified in the iCEL1314 model.

### WormPaths advantages

**Figure 3:**
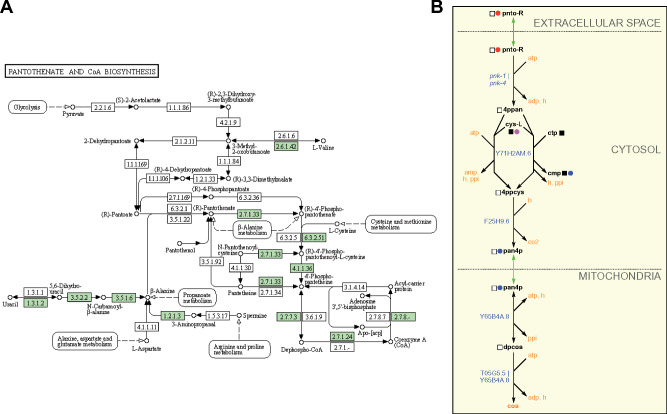
WormPaths provides easy to navigate *C. elegans*-specific maps. (A) Pantothenate and CoA biosynthesis metabolism map in KEGG. Green boxes indicate enzymes found in *C. elegans*. (B) Pantothenate and CoA biosynthesis map in WormPaths.

Metabolic maps provided by KEGG are extremely useful and frequently published in the primary literature (*e.g.*, Gao *et al.* 2018; Chan *et al.* 2019). However, these maps can be non-intuitive for several reasons. First, these ‘pan-organism’ maps display all the chemistry known for a particular pathway based on enzymes identified by Enzyme Commission number. However, many reactions can be found in some organisms but not others. For instance, many reactions are specific to prokaryotes. By selecting an organism of choice, here *C. elegans*, KEGG colors the boxes representing enzymes in green if the enzyme is predicted to occur in that organism (Figure 3A). Second, one has to hover over the enzyme box to visualize the associated gene(s). Third, there can be a lot of overlap between different pathways, and pathways in WormPaths have been greatly simplified without losing critical information (Figure 3B). For example, the *C. elegans* pantothenate and CoA biosynthesis map in KEGG looks extremely complicated, but many of the boxes in the KEGG map are white, indicating that there is no known gene for the pertaining reactions in *C. elegans*. Furthermore, the KEGG map contains components of cysteine and methionine metabolism, arginine and proline metabolism, propionate degradation, glycolysis, and other overlapping pathways. The WormPaths map strips away these excess genes and pathways and focuses solely on pantothenate and CoA formation (Figure 3B). In this specific example, connections to other pathways from the terminal metabolites are not indicated by boxes due to the fact that cys-L, ctp, cmp, and coa all connect to more than four other pathways, making the map cumbersome to navigate. The connecting pathways can be viewed on the WormFlux website by clicking the metabolite of interest.

**Figure 4:**
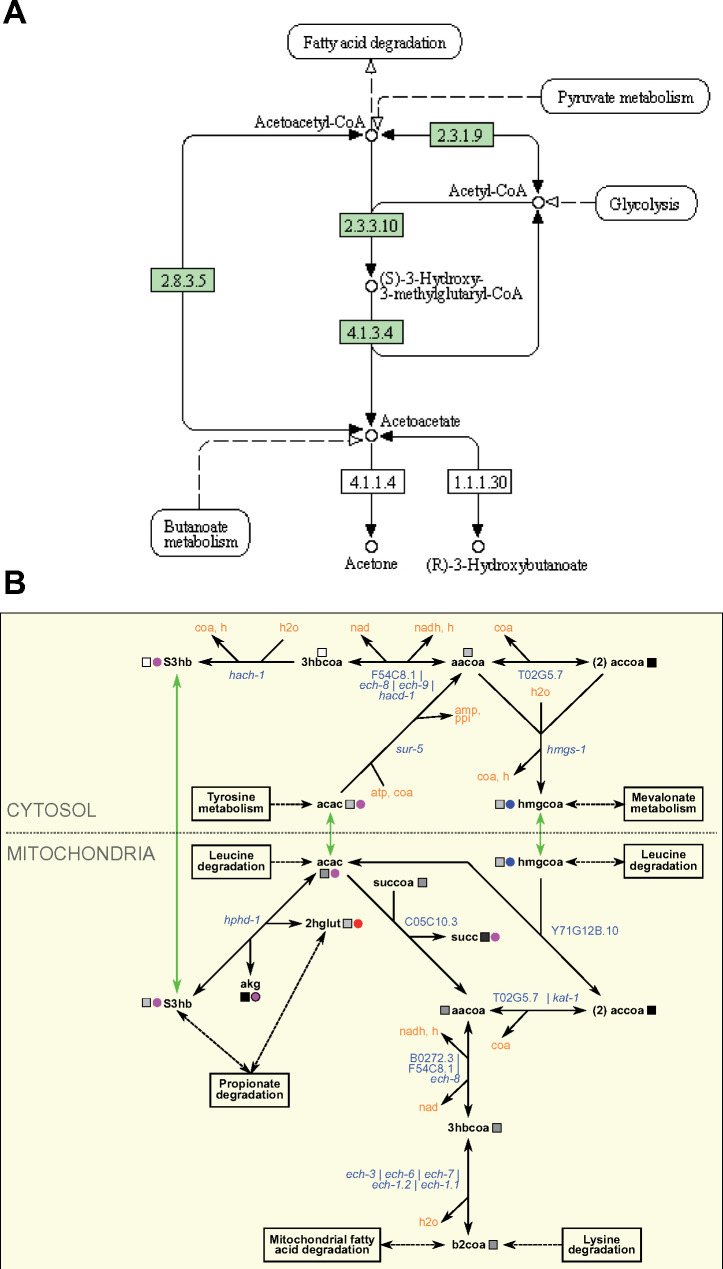
WormPaths maps provide additional reactions to metabolic pathways. (A) Ketone body metabolism map in KEGG. Green boxes indicate enzymes found in *C. elegans*. (B) Ketone body metabolism map in WormPaths.

In addition to simplifying metabolic pathway maps, we also extended several WormPaths maps relative to KEGG. For instance, the WormPaths ketone body metabolism map has additional conversions with associated genes, relative to the map available in KEGG (Figure 4). More precise connections to other pathways, transport reactions, and subcellular localization of the reactions are visualized in WormPaths.

**Figure 5:**
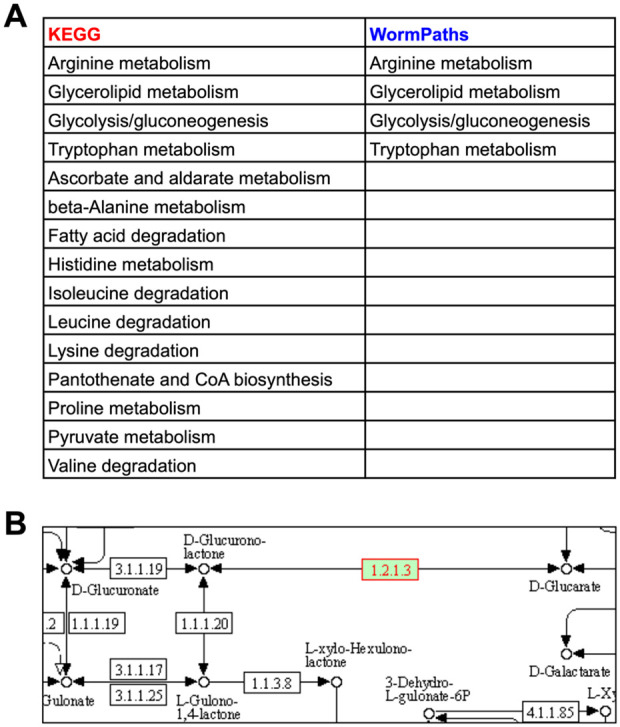
WormPaths maps clean up pathway associations for individual genes. (A) Gene-to-pathway annotations for *alh-2* in KEGG and WormPaths. (B) KEGG annotation for *alh-2* (green box with red text) in ascorbate and aldarate metabolism. White boxes indicate no known enzyme in *C. elegans*.

In KEGG, genes are associated with any pathway assigned to that gene by gene-protein-reaction associations. However, sometimes these reactions can be isolated because surrounding reactions are not found in the organism of interest, thus the isolated reaction does not connect to the larger pathway or network of said organism. The isolated reactions may be incorrect annotations that are not likely to exist in the organism, or they may have been incorrectly inserted into the pathway based on homology to another organism (Yilmaz and Walhout 2017). For instance, the aldehyde dehydrogenase *alh-2* is associated with 15 KEGG pathways (Figure 5A). However, in several of these KEGG reactions, *alh-2* is associated with one or more isolated reactions that are not connected to iCEL1314 (Yilmaz *et al.* 2020) (Figure 5B). This can be further visualized in the KEGG pantothenate and CoA biosynthesis map from Figure 3A; enzyme EC1.2.1.3 on the lower left is not connected to the rest of the pathway. Furthermore, only 5 of the 15 KEGG pathways associated with *alh-2* have the enzyme connected to the rest of the pathway via other *C. elegans* enzymes (glycolysis/gluconeogenesis, glycerolipid metabolism, leucine degradation, isoleucine degradation, and valine degradation). Altogether, WormPaths identifies four pathways for *alh-2*, and all are shared with KEGG (Figure 5A). Refining gene-to-pathway annotations in WormPaths is especially important for statistical analyses; when a gene is incorrectly associated with different pathways, this can affect the significance of detected enrichments.

### WormPaths levels can be used for pathway or gene set enrichment analysis

To demonstrate how the levels in WormPaths can be used to identify high-resolution metabolic pathway enrichment in transcriptomic data, we analyzed a previously published RNA-seq dataset measuring the transcriptomes of untreated animals, animals treated with 20 nM vitamin B12, or 20 nM vitamin B12 and 40 mM propionate (Bulcha *et al.* 2019). We selected all of the genes from the genome scale experiment with a *p*-adjusted value of ≤0.05 and a fold change of ±1.5 and performed PEA using the PEA tool in WormFlux (see Materials and methods and Supplementary Figure S3). This approach confirmed our previous findings that propionate degradation by the shunt pathway and the Met/SAM cycle are enriched in this dataset (Bulcha *et al.* 2019; Giese *et al.* 2020).

**Figure 6:**
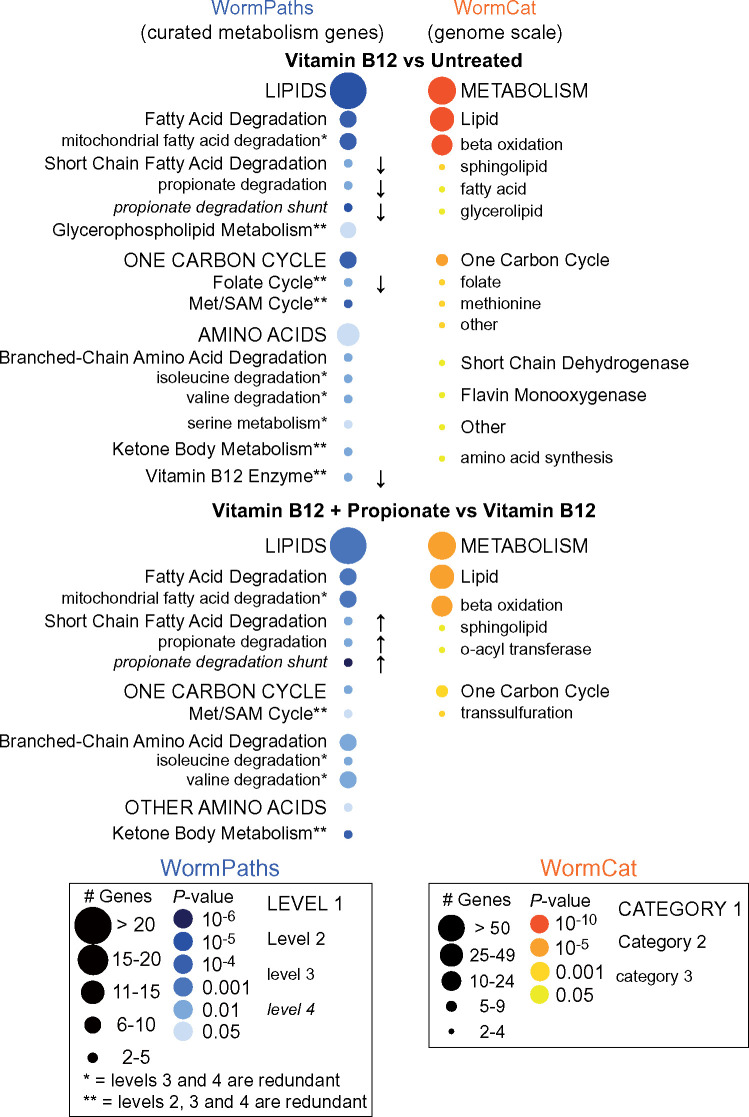
Pathway enrichment analysis using WormPaths levels. Pathway enrichment analysis using a previously published RNA-seq dataset of *C. elegans* untreated, treated with 20nM vitamin B12, or treated with 20nM vitamin B12 and 40mM propionate with a *p*-adjusted of ≤0.05 and a fold change of ±1.5 shows enrichment of lipids and one-carbon cycle pathways (left, blue). The arrows indicate the directionality of differentially expressed genes. No arrow indicates both increased and decreased gene expression. WormPaths enrichment for curated metabolic genes complements and adds resolution to the genome scale enrichment metabolic results from WormCat (right, orange).

In collaboration with the Walker lab, we previously developed WormCat, an online tool for identifying genome-scale coexpressed gene sets (Holdorf *et al.* 2019) (Figure 6). In WormCat, genes are assigned to a single functional annotation, while in WormPaths, metabolic genes can be assigned to multiple reactions and, therefore, pathways. This, together with the inclusion of different Levels of metabolism, allows gene enrichment analysis at greater resolution (Figure 6). In contrast to WormCat, however, WormPaths is limited to the genes included in the iCEL1314 model (Yilmaz *et al.* 2020). Given the advanced curation of the genes in WormPaths, using these gene sets provides a complementary level of resolution for the analysis of metabolic pathways, relative to WormCat. Thus, we suggest that researchers first use WormCat for gene set enrichment analysis and that they include WormPaths in their analyses when they find an enrichment for metabolic genes. Finally, our high-resolution metabolic pathway annotations can be integrated as custom gene-sets while performing other kinds of enrichment analysis, for example using classical Gene Set Enrichment Analysis (Subramanian *et al.* 2005) to extract specific desired information from gene expression profiling data.

## Discussion and vision

We have developed WormPaths, an expandable online catalog of *C. elegans* metabolic pathway maps and gene annotations. Our overall annotations predict a total of more than 3000 metabolic genes in *C. elegans*, based on homologies with metabolic enzymes or protein domains (Yilmaz and Walhout 2016). Therefore, metabolic network models such as iCEL1314 continue to grow and evolve as more experimental data becomes available. We encourage *C. elegans* researchers to contact us and help with updates and additions, and to point out any errors they may find. We expect that new metabolic reactions and metabolites will continue to be added to future versions of iCEL as they are discovered. For instance, the iCEL1314 model incorporates the relatively recently discovered ascaroside biosynthesis pathway (Yilmaz *et al.* 2020). In the future, we hope to visualize changes in gene expression, metabolite concentrations, and potentially metabolic rewiring, which can occur under different dietary or environmental conditions. Altogether, WormPaths builds on and provides advantages over KEGG and the visualization strategy used to develop WormPaths should be applicable to other model organisms.

## Supplementary Material

iyab089_Supplementary_Data

## References

[iyab089-B1] Bulcha JT, Giese GE, Ali MZ, Lee Y-U, Walker M, et al 2019. A persistence detector for metabolic network rewiring in an animal. Cell Rep. 26:460–468. 30625328 10.1016/j.celrep.2018.12.064 PMC6368391

[iyab089-B2] Cao J, Packer JS, Ramani V, Cusanovich DA, Huynh C, et al 2017. Comprehensive single-cell transcriptional profiling of a multicellular organism. Science. 357:661–667. 28818938 10.1126/science.aam8940 PMC5894354

[iyab089-B3] Caspi R, Altman T, Billington R, Dreher K, Foerster H, et al 2014. The MetaCyc database of metabolic pathways and enzymes and the BioCyc collection of Pathway/Genome Databases. Nucleic Acids Res. 42:D459–D471. 24225315 10.1093/nar/gkt1103 PMC3964957

[iyab089-B4] Chan JP, Wright JR, Wong HT, Ardasheva A, Brumbaugh J, et al 2019. Using bacterial transcriptomics to investigate targets of host-bacterial interactions in *Caenorhabditis elegans*. Sci Rep. 9:5545. 30944351 10.1038/s41598-019-41452-2 PMC6447554

[iyab089-B5] Chang A, Schomburg I, Placzek S, Jeske L, Ulbrich M, et al 2015. BRENDA in 2015: exciting developments in its 25th year of existence. Nucleic Acids Res. 43:D439–D446. 25378310 10.1093/nar/gku1068 PMC4383907

[iyab089-B6] Coolon JD, Jones KL, Todd TC, Carr BC, Herman MA. 2009. *Caenorhabditis elegans* genomic response to soil bacteria predicts environment-specific genetic effects on life history traits. PLoS Genet. 5:e1000503. 19503598 10.1371/journal.pgen.1000503 PMC2684633

[iyab089-B7] Corsi AK, Wightman B, Chalfie M. 2015. A transparent window into biology: a primer on *Caenorhabditis elegans*. Genetics. 200:387–407. 26088431 10.1534/genetics.115.176099 PMC4492366

[iyab089-B8] Dalby A, Nourse JG, Hounshell WD, Gushurst AKI, Grier DL, et al 1992. Description of several chemical structure file formats used by computer programs developed at Molecular Design Limited. J Chem Inf Comput Sci. 32:244–255.

[iyab089-B9] Gao AW, Smith RL, van Weeghel M, Kamble R, Janssens GE, et al 2018. Identification of key pathways and metabolic fingerprints of longevity in *C. elegans*. Exp Gerontol. 113:128–140. 30300667 10.1016/j.exger.2018.10.003 PMC6224709

[iyab089-B10] Giese GE, Walker MD, Ponomarova O, Zhang H, Li X, et al 2020. *C. elegans* methionine/S-adenosylmethionine cycle activity is sensed and adjusted by a nuclear hormone receptor. Elife. 9:e60259. 33016879 10.7554/eLife.60259 PMC7561351

[iyab089-B11] Gusarov I, Gautier L, Smolentseva O, Shamovsky I, Eremina S, et al 2013. Bacterial nitric oxide extends the lifespan of *C. elegans*. Cell. 152:818–830. 23415229 10.1016/j.cell.2012.12.043

[iyab089-B12] Harris TW, Arnaboldi V, Cain S, Chan J, Chen WJ, et al 2020. WormBase: a modern model organism information resource. Nucleic Acids Res. 48:D762–D767. 31642470 10.1093/nar/gkz920 PMC7145598

[iyab089-B13] Holdorf AD, Higgins DP, Hart AC, Boag PR, Pazour GJ, et al 2019. WormCat: an online tool for annotation and visualization of *Caenorhabditis elegans* genome-scale data. Genetics. 214:279–294. 31810987 10.1534/genetics.119.302919 PMC7017019

[iyab089-B14] Joshi-Tope G, Gillespie M, Vastrik I, D'Eustachio P, Schmidt E, et al 2005. Reactome: a knowledgebase of biological pathways. Nucleic Acids Res. 33:D428–D432. 15608231 10.1093/nar/gki072 PMC540026

[iyab089-B15] Kanehisa M, Sato Y, Kawashima M, Furumichi M, Tanabe M. 2015. KEGG as a reference resource for gene and protein annotation. Nucleic Acids Res. 44:D457–D462. 26476454 10.1093/nar/gkv1070 PMC4702792

[iyab089-B16] Larsen PL, Clarke CF. 2002. Extension of life-span in *Caenorhabditis elegans* by a diet lacking coenzyme Q. Science. 295:120–123. 11778046 10.1126/science.1064653

[iyab089-B17] Lemieux GA, Ashrafi K. 2016. Investigating connections between metabolism, longevity, and behavior in *Caenorhabditis elegans*. Trends Endocrinol Metab. 27:586–596. 27289335 10.1016/j.tem.2016.05.004 PMC4958586

[iyab089-B18] Machado D, Herrgard M. 2014. Systematic evaluation of methods for integration of transcriptomic data into constraint-based models of metabolism. PLoS Comput Biol. 10:e1003580. 24762745 10.1371/journal.pcbi.1003580 PMC3998872

[iyab089-B19] MacNeil LT, Walhout AJM. 2013. Food, pathogen, signal: the multifaceted nature of a bacterial diet. Worm. 2:e26454. 24744980 10.4161/worm.26454 PMC3917966

[iyab089-B20] MacNeil LT, Watson E, Arda HE, Zhu LJ, Walhout AJM. 2013. Diet-induced developmental acceleration independent of TOR and insulin in *C. elegans*. Cell. 153:240–252. 23540701 10.1016/j.cell.2013.02.049 PMC3821073

[iyab089-B21] Nigon VM, Felix MA. 2017. History of research on *C. elegans* and other free-living nematodes as model organisms. WormBook. 2017:1–84. 10.1895/wormbook.1.181.1 PMC5611556 28326696

[iyab089-B22] Noronha A, Modamio J, Jarosz Y, Guerard E, Sompairac N, et al 2019. The Virtual Metabolic Human database: integrating human and gut microbiome metabolism with nutrition and disease. Nucleic Acids Res. 47:D614–D624. 30371894 10.1093/nar/gky992 PMC6323901

[iyab089-B23] O'Boyle NM, Banck M, James CA, Morley C, Vandermeersch T, et al 2011. Open Babel: an open chemical toolbox. J Cheminform. 3:33. 21982300 10.1186/1758-2946-3-33 PMC3198950

[iyab089-B24] Opdam S, Richelle A, Kellman B, Li S, Zielinski DC, et al 2017. A systematic evaluation of methods for tailoring genome-scale metabolic models. Cell Syst. 4:318–329.e6. 28215528 10.1016/j.cels.2017.01.010 PMC5526624

[iyab089-B25] Rashid S, Pho KB, Mesbahi H, MacNeil LT. 2020. Nutrient sensing and response drive developmental progression in *Caenorhabditis elegans*. Bioessays. 42:e1900194. 32003906 10.1002/bies.201900194

[iyab089-B26] Schellenberger J, Park JO, Conrad TM, Palsson BO. 2010. BiGG: a Biochemical Genetic and Genomic knowledgebase of large scale metabolic reconstructions. BMC Bioinformatics. 11:213. 20426874 10.1186/1471-2105-11-213 PMC2874806

[iyab089-B27] Subramanian A, Tamayo P, Mootha VK, Mukherjee S, Ebert BL, et al 2005. Gene set enrichment analysis: a knowledge-based approach for interpreting genome-wide expression profiles. Proc Natl Acad Sci USA. 102:15545–15550. 16199517 10.1073/pnas.0506580102 PMC1239896

[iyab089-B28] Virk B, Jia J, Maynard CA, Raimundo A, Lefebvre J, et al 2016. Folate acts in *E. coli* to accelerate *C. elegans* aging independently of bacterial biosynthesis. Cell Rep. 14:1611–1620. 26876180 10.1016/j.celrep.2016.01.051 PMC4767678

[iyab089-B29] Watson E, MacNeil LT, Arda HE, Zhu LJ, Walhout AJM. 2013. Integration of metabolic and gene regulatory networks modulates the *C. elegans* dietary response. Cell. 153:253–266. 23540702 10.1016/j.cell.2013.02.050 PMC3817025

[iyab089-B30] Watson E, MacNeil LT, Ritter AD, Yilmaz LS, Rosebrock AP, et al 2014. Interspecies systems biology uncovers metabolites affecting *C. elegans* gene expression and life history traits. Cell. 156:759–770. 24529378 10.1016/j.cell.2014.01.047 PMC4169190

[iyab089-B31] Watson E, Olin-Sandoval V, Hoy MJ, Li C-H, Louisse T, et al 2016. Metabolic network rewiring of propionate flux compensates vitamin B12 deficiency in *C. elegans*. Elife. 5:e17670. 27383050 10.7554/eLife.17670 PMC4951191

[iyab089-B32] Watson E, Walhout AJ. 2014. *Caenorhabditis elegans* metabolic gene regulatory networks govern the cellular economy. Trends Endocrinol Metab. 25:502–508. 24731597 10.1016/j.tem.2014.03.004 PMC4178166

[iyab089-B33] Watts JL. 2016. Using *Caenorhabditis elegans* to uncover conserved functions of omega-3 and omega-6 fatty acids. J Clin Med. 5:19. 10.3390/jcm5020019 PMC4773775 26848697

[iyab089-B34] Yilmaz LS, Li X, Nanda S, Fox B, Schroeder F, et al 2020. Modeling tissue-relevant *Caenorhabditis elegans* metabolism at network, pathway, reaction, and metabolite levels. Mol Syst Biol. 16:e9649. 33022146 10.15252/msb.20209649 PMC7537831

[iyab089-B35] Yilmaz LS, Walhout AJ. 2016. A *Caenorhabditis elegans g*enome-scale metabolic network model. Cell Syst. 2:297–311. 27211857 10.1016/j.cels.2016.04.012 PMC5387690

[iyab089-B36] Yilmaz LS, Walhout AJ. 2017. Metabolic network modeling with model organisms. Curr Opin Chem Biol. 36:32–39. 28088694 10.1016/j.cbpa.2016.12.025 PMC5458607

[iyab089-B37] Yilmaz LS, Walhout AJM. 2014. Worms, bacteria and micronutrients: an elegant model of our diet. Trends Genet. 30:496–503. 25172020 10.1016/j.tig.2014.07.010 PMC4399232

[iyab089-B38] Zhang J, Li X, Olmedo M, Holdorf AD, Shang Y, et al 2019. A delicate balance between bacterial iron and reactive oxygen species supports optimal *C. elegans* development. Cell Host Microbe. 26:400–411.e3. 31444089 10.1016/j.chom.2019.07.010 PMC6742550

