# Supplementary material for: WormPaths: Caenorhabditis elegans metabolic pathway annotation and visualization
Source: Genetics. 2021 Jun 12;219(1):iyab089. doi: 10.1093/genetics/iyab089 (PMC8864737; doi:10.1093/genetics/iyab089)
Supplement: iyab089_Supplementary_Data [file iyab089_Supplementary_Data.zip › GENETICS-GENETICS-2021-304284-s01.pdf]

WormPaths Supplementary Figure S1

Irreversible Reactions

Edge	#reactants	#products	side metabolites
	1	1	No
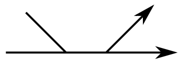	1	1	Yes
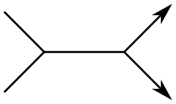	2	2	No
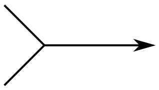	2	1	No
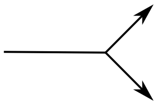	1	2	No
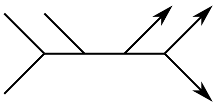	2	2	Yes
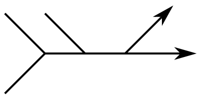	2	1	Yes
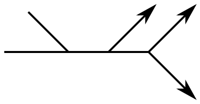	1	2	Yes

Reversible Reactions

Edge	#reactants	#products	side metabolites
	1	1	No
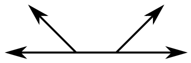	1	1	Yes
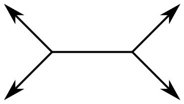	2	2	No
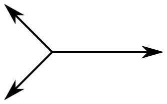	2	1	No
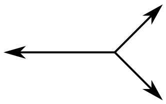	1	2	No
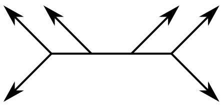	2	2	Yes
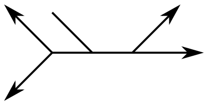	2	1	Yes
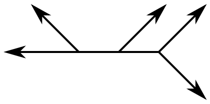	1	2	Yes

Main Metabolites

accoa
akg
cys-L
.
.
.
.
.

Co-reactants

adp	gdp
amp	gtp
atp	h
co2	h2o
coa	nad
crn	nadh
etfox	nadp
etfrd	nadph
fad	o2
fadh2	pi
	ppi

GPR

acdh-1
idh-1 | idh-2
sucl-1 | sucl-2 & suca-1
.
.
.
.
.
