# Supplementary material for: WormPaths: Caenorhabditis elegans metabolic pathway annotation and visualization
Source: Genetics. 2021 Jun 12;219(1):iyab089. doi: 10.1093/genetics/iyab089 (PMC8864737; doi:10.1093/genetics/iyab089)
Supplement: iyab089_Supplementary_Data [file iyab089_Supplementary_Data.zip › GENETICS-GENETICS-2021-304284-s06.docx]

**SUPPLEMENTARY MATERIAL**

**Figure S1. Template used for drawing WormPaths maps**

GPR, gene-protein reaction association

**Figure S2. Formatting annotations and other design information for WormPaths maps**

GPR, gene-protein reaction association

**Figure S3. Workflow for using pathway enrichment analysis in WormFlux**

A. The main screen of the pathway enrichment analysis tool (PEA, highlighted in yellow on the left). Data from the previously published RNA-seq dataset used in Figure 6 is entered as WormBase IDs in the “Input” box.

B. After pressing “SUBMIT” the results are displayed at all levels regardless of enrichment or depletion. Enriched pathways are highlighted in darkening shades of magenta, and *p*-values are represented as colored borders on the pathways at each level, with lower *p*-values having thicker borders. Border colors for the cases of enrichment and depletion are orange and dark blue, respectively.

C. The key to the enrichment table.

D. Hovering the cursor over a pathway shows the number of hits in the pathway and the *p*-value.

E. Clicking on the pathway brings up the pathway map with the genes from the entered dataset highlighted in grey.

F. Downloadable tab-separated text file of all category enrichment and depletion data.

**Figure S4. An example of using WormPaths to search for a specific gene**

A search for the gene *metr-1* will lead to the gene overview, followed by the specific pathway maps that *metr-1* is involved in with the gene highlighted in red.

**Figure S5. Tissue-specific expression of *pck-1* and *pck-2***

**Table S1. Pathways at levels 1 through 4**

**Table S2. All maps and the corresponding level to which each map was drawn**

**Table S3. Gene sets per each pathway by level by gene name**

**Table S4. Gene sets per each pathway by level by WormBase ID**

**Table S5. All pathway associations listed by gene**
