# Supplementary figures and images for: WormPaths: Caenorhabditis elegans metabolic pathway annotation and visualization
Source: Genetics. 2021 Jun 12;219(1):iyab089. doi: 10.1093/genetics/iyab089 (PMC8864737; doi:10.1093/genetics/iyab089)

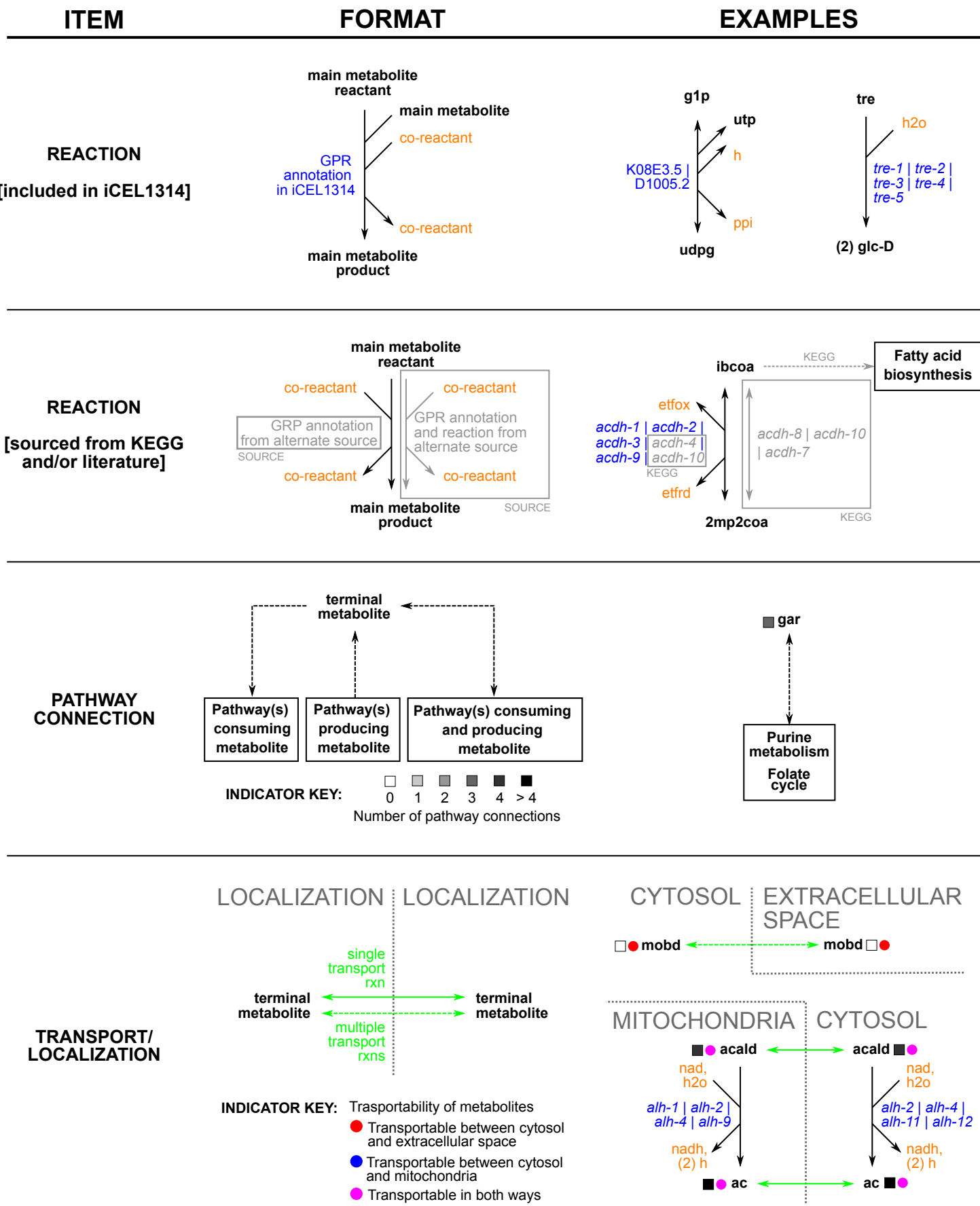

Supplement: iyab089_Supplementary_Data [file iyab089_Supplementary_Data.zip › GENETICS-GENETICS-2021-304284-s02.pdf]

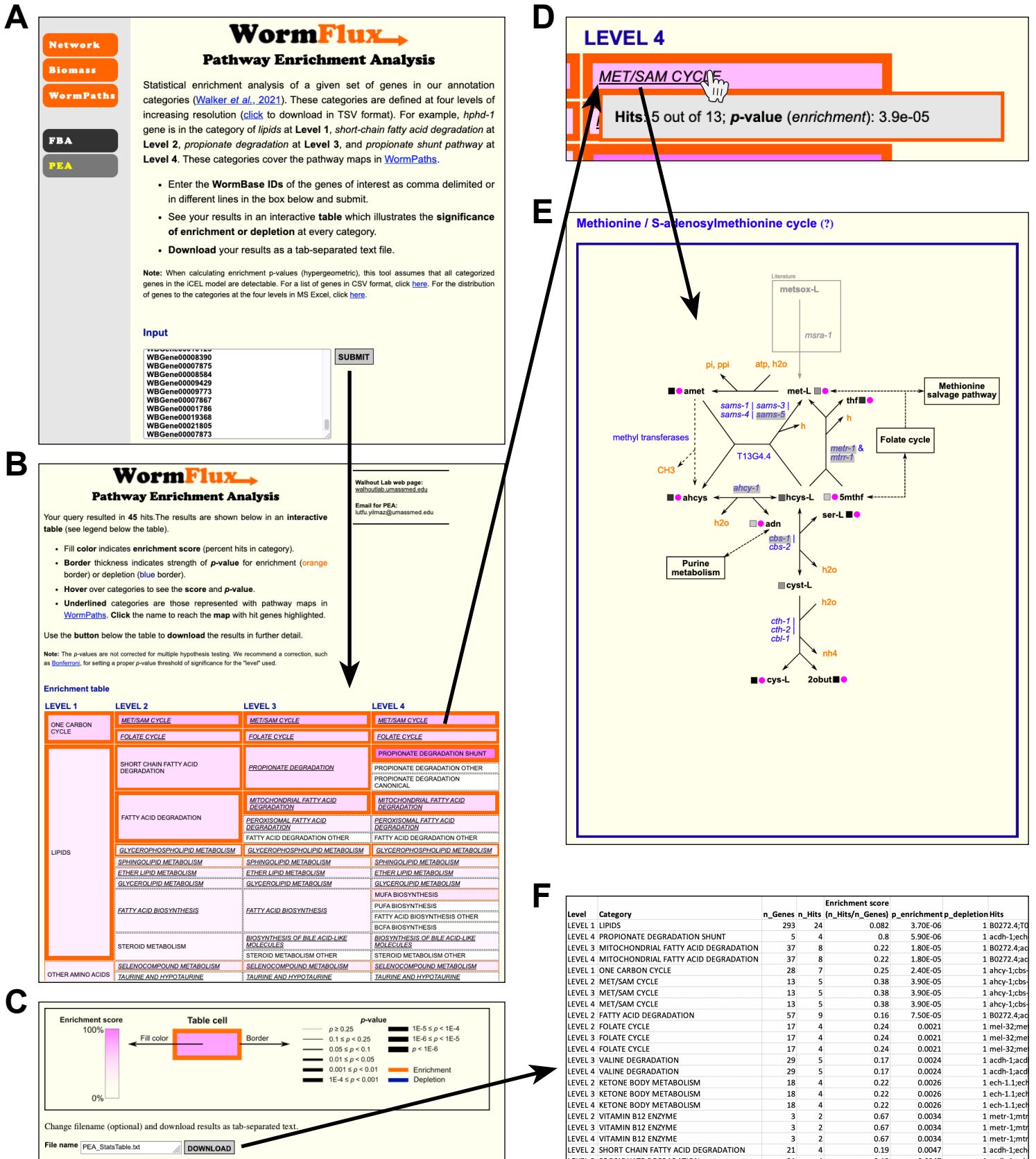

Supplement: iyab089_Supplementary_Data [file iyab089_Supplementary_Data.zip › GENETICS-GENETICS-2021-304284-s03.pdf]

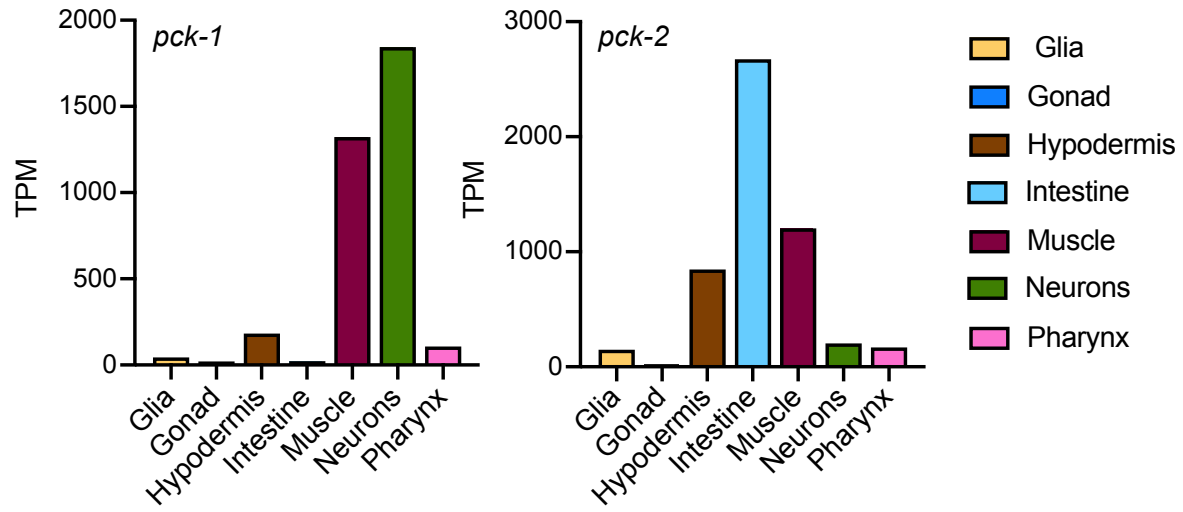

Supplement: iyab089_Supplementary_Data [file iyab089_Supplementary_Data.zip › GENETICS-GENETICS-2021-304284-s05.pdf]
